# K-edge subtraction imaging for coronary angiography with a compact synchrotron X-ray source

**DOI:** 10.1371/journal.pone.0208446

**Published:** 2018-12-10

**Authors:** Stephanie Kulpe, Martin Dierolf, Eva Braig, Benedikt Günther, Klaus Achterhold, Bernhard Gleich, Julia Herzen, Ernst Rummeny, Franz Pfeiffer, Daniela Pfeiffer

**Affiliations:** 1 Chair of Biomedical Physics, Department of Physics, Technical University of Munich, Garching, Germany; 2 Munich School of BioEngineering, Technical University of Munich, Garching, Germany; 3 Department of Diagnostic and Interventional Radiology, Klinikum rechts der Isar, Technical University of Munich, Munich, Germany; Emory University School of Medicine, UNITED STATES

## Abstract

About one third of all deaths worldwide can be traced back to cardiovascular diseases. An interventional radiology procedure for their diagnosis is Digital Subtraction Angiography (DSA). An alternative to DSA is K-Edge subtraction (KES) imaging, which has been shown to be advantageous for moving organs and eliminating image artifacts caused by patient movement. As highly brilliant, monochromatic X-rays are required for this method, it has been limited to synchrotron facilities so far, restraining the feasibility in clinical routine. Compact synchrotron X-ray sources based on inverse Compton scattering, which have been evolving substantially over the past decade, provide X-rays with sufficient brilliance that meet spatial and financial requirements affordable in laboratory settings or for university hospitals. In this work, we demonstrate a first proof-of-principle K-edge subtraction imaging experiment using the Munich Compact Light Source (MuCLS), the first user-dedicated installation of a compact synchrotron X-ray source worldwide. It is shown experimentally that the technique of KES increases the visibility of small blood vessels overlaid by bone structures.

## Introduction

Digital Subtraction Angiography (DSA) is one of the most important examinations in the diagnosis of cardiovascular disease and treatment of blood vessels [1–3]. It is highly effective in enhancing contrast between vascular structures and surrounding soft tissue as well as bone. However, motion artifacts caused by patient motion, respiration and cardiac motion limit the application of this method. The resulting artifacts are particularly severe when imaging coronary arteries [4].

K-edge subtraction (KES) imaging exploits the sharp increase of the absorption coefficient of a contrast agent to acquire images at energies just below and above the K-edge energy. In contrast to DSA, where two X-ray images are taken before and after the injection of the iodine contrast agent, images in KES imaging are both taken after the injection, but at different X-ray energies. Elleaume et al. [5] showed that this makes the method more suitable for imaging moving organs. However, as highly brilliant X-rays are required for this imaging method, it has been limited to synchrotron facilities so far. Rubenstein et al. [6,7] were the first to describe a dual-energy subtraction technique using synchrotron radiation, imaging coronary arteries around the K-edge of iodine. Elleaume et al. [8] showed that it is possible to acquire high quality images at a synchrotron for coronary angiography in patients after intravenous injection of iodine contrast agent. A comparison in [9] of dual energy KES-imaging with conventional X-ray spectra and KES-imaging with monochromatic X-rays demonstrated improved quality for images of the neurovascular system when imaging with monochromatic X-rays. Especially for small vessels that could be blocked by a catheter used for injection, this small-animal study concluded that intravenous injection combined with the KES method at a synchrotron lowers patient risk while achieving high quality images. To enable KES without changing the X-ray energy of the synchrotron source Umetani et al. [10,11] suggested the use of an X-ray filter that partially absorbs the X-ray spectrum and thereby changes its mean energy.

While synchrotrons provide highly brilliant, monochromatic X-rays, they rely on electron storage rings of several hundred meters in circumference and are expensive in terms of installation as well as operation and maintenance. In contrast, the use of conventional X-ray tubes in laboratories and hospitals is comparably cheap, but they feature low brilliance and polychromatic X-ray spectra. A conventional X-ray spectrum will be modified as it traverses the patient, since lower energies are attenuated more strongly than higher ones. This beam hardening impairs the measurement accuracy and can interfere with the goal to optimize both dose level and image quality. As K-edge subtraction imaging needs a monochromatic X-ray beam, its feasibility in clinical routine has been limited. Compact synchrotron X-ray sources provide brilliance in between those of a large-scale synchrotron and a conventional X-ray source. The Munich Compact Light Source (MuCLS) provides high-intensity X-rays that are quasi-monochromatic and are emitted into a much smaller opening angle than at an X-ray tube, thereby providing a brilliance of ~5 x 10^9^ (at 25 keV, [12]) in comparison to a rotating anode with ~0.6 x 10^9^ [13] and a third generation synchrotron ~10^21^ [14,15]. Compared to the latter, a main advantage of compact synchrotron sources are the reduced spatial and financial requirements [12,16] for acquisition, operation and maintenance. These lower investment costs enable the transfer of certain techniques that have been limited to synchrotrons so far, like KES-imaging, into a laboratory or pre-clinical environment. It has been shown previously by Eggl et al. [17] that image acquisition in coronary angiography is possible with higher contrast-to-noise ratio (CNR) between blood vessels and surrounding tissue at the MuCLS than at a conventional X-ray source, if an energy is used that lies directly at the K-edge of the iodine contrast agent. It was shown that the dose can be potentially reduced when imaging with a monochromatic spectrum due to the restriction to photon energies with an optimum contrast-to-dose ratio. The absence of low energy photons that are usually responsible for a large amount of the dose, together with the ability to tune the X-ray source to the K-edge of the contrast agent, thereby reduces the applied dose and at the same time improves image contrast. However, in those experiments, the heart was considered as an isolated object on a flat background. In a realistic setting, the coronary arteries will be overlaid by bone and other tissue structures, which compromises the visibility of the vessels. Therefore, we propose the use of KES to eliminate overlying structures from the images. As the energy of the MuCLS cannot be changed without modifying many parameters, the filtering approach developed at a synchrotron by Umetani et al. [10,11] was implemented at the MuCLS.

Here, we present a KES-imaging approach for coronary angiography with filter-based KES-imaging at a compact synchrotron source. The filtering approach was implemented to enable a fast change in X-ray energy and thus enable dynamic KES imaging for future in-vivo experiments. The results of our experiments show that KES-imaging, in combination with a compact synchrotron X-ray source that is tuned to the K-edge of the contrast medium, can enhance the contrast and visibility of blood vessels lying behind bone structures.

## Materials and methods

### Working principle of the MuCLS

The Munich Compact Light Source (MuCLS) was developed and manufactured by Lyncean Technologies Inc., USA. It is a compact synchrotron source based on inverse Compton scattering and produces X-rays through the collision of relativistic electrons with infrared laser photons, thereby providing a quasi-monoenergetic, tunable X-ray beam [12,16]. In the collision of a highly relativistic electron with a laser photon, an X-ray photon with an energy

$$E_{x}\approx 4\gamma^{2}E_{L}$$ (1)

is produced, where *E*_*L*_ is the laser photon energy, *γ* = *E*_*e*_/*mc*^2^ is the ratio of electron energy to electron rest energy, and the approximation assumes head-on collision of laser photon and electron as well as backscattering of the X-ray photon [18]. In order to ensure a continuously high X-ray flux, the laser pulse is stored in a high finesse optical resonator while the electrons orbit in a storage ring. Their revolution frequencies are matched so that they collide at the interaction point upon each revolution. The X-rays are emitted into an opening angle of 4 mrad. The X-ray beam is quasi-monochromatic with a bandwidth ΔE/E below 4.5% full width at half maximum (FWHM) and partially coherent. By adjusting the electron energy, the X-ray energy is tunable between 15 and 35 keV. At the time of the measurements, the MuCLS produced a flux of up to 1.2 x 10^10^ photons per second with a source size of approximately 45 x 45 μm^2^. After our measurements, the machine was upgraded and can now provide about twice the X-ray flux [19].

**Fig 1:**
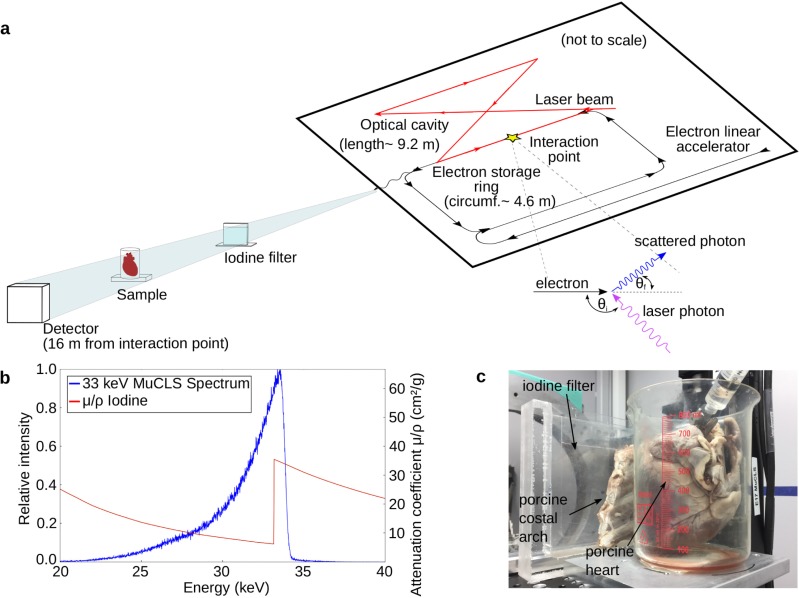
**(a)** Schematic of the experimental set-up at the Munich Compact Light Source (MuCLS): X-rays are produced at the interaction point of laser photons stored in the optical cavity and relativistic electrons circulating in the storage ring. These propagate in a vacuum tube to the experiment at a source-to sample distance of 15.5 m. The detector is located at a distance of 16 m from the interaction point. **(b)** X-ray spectrum with the peak energy tuned to 33.49 keV measured with a KETEK AXAS-D detector, shown together with the iodine attenuation coefficient. **(c)** Photograph of the experimental set-up showing the iodine filter in front of a porcine coastal arch and a porcine heart.

The X-ray experiments were performed at a dedicated imaging beam line, developed and installed by TUM, featuring two end stations (near and far). The spectral measurements were performed in the first end station at about 5 m from the interaction point. The X-ray beam travels partially through an evacuated beam pipe on its way from the source to the detector in the far end station. The spectral measurements and considerations for the spectra at the sample-to-detector distance were corrected for the effects of the windows of the vacuum beam pipe and air absorption. For all other experiments, the far end station was used, with the sample placed at a distance of about 15.5 m from the interaction point, where the beam has an elliptic extent of 62 x 74 mm^2^. A schematic of the MuCLS and the attached beam line infrastructure is shown in Fig 1A, together with a photo of the sample setup (Fig 1C).

### K-edge subtraction imaging at the MuCLS

As the energy tuning of the MuCLS presently takes approximately an hour, the much faster filter approach was implemented to perform KES imaging at the MuCLS. Fig 1B shows the unfiltered spectrum of the MuCLS with a peak energy of 33.49 keV measured with a KETEK AXAS-D detector–optimized for KES at the iodine K-edge–together with the iodine absorption within this energy range.

A series of three experiments were performed to demonstrate the feasibility of the proposed KES technique. First, a phantom was constructed of an empty acrylic glass container with plates of aluminum mounted in front of a tube filled with undiluted iodine-based contrast agent (IMERON 400 MCT, Bracco Imaging, Germany). Aluminum is a material with absorption properties similar to bone [20,21]. This phantom mimics a bone in front of an iodine contrasted blood vessel, as it is commonly the case in clinical applications. A series of experiments with different thicknesses of aluminum was performed to evaluate how the CNR between iodine and aluminum changes with increasing thickness. The aluminum thicknesses were 0.1 cm, 0.2 cm and 0.5 cm, corresponding to bone thicknesses of 0.11 cm, 0.23 cm and 0.56 cm, respectively [22]. Second, an excised porcine heart was placed into a plastic beaker glass and undiluted iodine-based contrast agent was injected via catheter into the onset of the coronary artery. To simulate the situation in a realistic setting, namely ribs of the chest overlying the coronary arteries, a porcine costal arch fixated in formalin was set in front of the heart in the experimental set-up (see Fig 1C) in a final step. All samples were positioned at a source-to-sample distance of 15.5 m. The images were acquired with a flat panel detector (Dexela 1512, PerkinElmer, Inc., USA) placed at a distance of 16 meters from the source point. It is equipped with a Gd_2_O_2_S scintillator and has a pixel size of 74.8 x 74.8 μm^2^. The effective pixel size of the acquired images was 70 x 70 μm^2^. As the pixel size for coronary angiography in a clinical procedure is typically around 200 μm [23,24] (German guidelines for coronary angiography recommend a range of 200–500 μm [25]), the acquired images were binned with a factor of 3 to pixel sizes of 210 x 210 μm^2^. Two images of each sample were taken, the first one with the native spectrum, the second one filtered with an iodine filter. The iodine filter was made of an acrylic glass container filled with iodine solution exhibiting a thickness of 2 mm along the beam direction and an iodine content of 400 mg/ml (IMERON 400 MCT, Bracco Imaging, Germany), amounting to an effective iodine thickness of around 150 μm. The iodine filter was constructed such that a large part of the spectrum above the K-edge of iodine was absorbed (~3% of the original intensity remaining) while maintaining a large part of the low energy part of the spectrum (~48% of the original intensity remaining). The switching of the filter was done manually. Exposure times of the images were adjusted to account the for lower photon flux due to filter absorption in the iodine-filtered images, being 1 s for the unfiltered image and 2 s for the iodine-filtered image. Accordingly, separate reference (empty-beam) images for both settings (filter/no filter) were recorded too. With these, a reference correction of the filtered and the unfiltered image is performed before any other processing step. When applying a filter approach to KES imaging, it is necessary to post-process the measured data further in order to obtain two images with mean energies below and above the K-edge of the contrast agent using a procedure described in [10,11].

**Fig 2:**
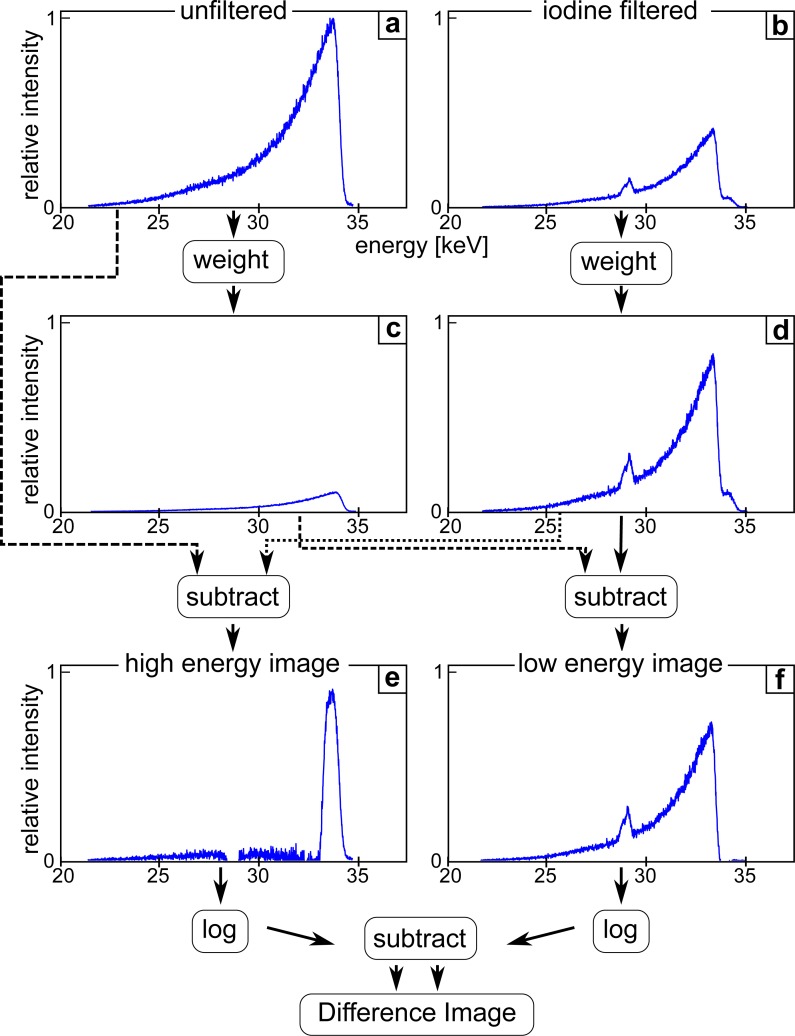
Workflow used for calculating a K-edge subtraction image at the MuCLS. First, unfiltered and iodine-filtered images are recorded and dark current, flatfield and flux corrected. To obtain two images only containing the high- or low-energy part of the spectrum, one needs to eliminate the high-energy part in the iodine filtered image and the low-energy part in the unfiltered image. The iodine-filtered image is weighted so that its intensity corresponds to the low-energy part of the unfiltered image, which results in the weighted iodine-filtered image **(d)**. Subtracting image **(d)** from the unfiltered image **(a)**, the high-energy image **(e)** is obtained, as the low-energy parts of the spectra are identical and fall away in the subtraction. To obtain the low-energy image, the high-energy part of the weighted iodine-filtered image has to be eliminated. Therefore, the unfiltered image **(a)** is weighted so that it corresponds to the remaining high-energy part of the weighted iodine-filtered image. The weighted unfiltered image **(c)** is then subtracted from the weighted iodine-filtered image **(d)**, yielding a low-energy image **(f)**, where the high-energy contribution is completely cancelled out. Finally, the difference image is obtained by logarithmically subtracting the low-energy image from the high-energy one.

This workflow to calculate the two images is shown in Fig 2 by illustrating the effect of the different processing steps on the X-ray spectrum which contributes to the respective image. By first determining the steps and scaling factors required to obtain a high-energy and a low-energy spectrum without any remaining overlap, the same procedure can then be applied to the filtered and unfiltered image. For this purpose, two spectra have been recorded with a silicon drift-detector (AXAS-D, KETEK) at identical settings, one with the iodine filter and one without the filter. The small peak in the iodine-filtered spectrum is the iodine fluorescence of the filter which does not influence the image quality of the acquired images due to a larger filter-detector distance in the experimental setup. To obtain two images containing only the high-energy or the low-energy part of the spectrum, respectively, the high-energy part in the iodine-filtered image has to be eliminated as well as the low-energy part in the unfiltered image. Therefore, the iodine-filtered spectrum Fig 2B is weighted such that its intensity corresponds to the one of the low-energy part of the unfiltered spectrum. By matching the slope of the filtered spectrum to the unfiltered one, the fraction *a* of the filtered spectrum contained in the unfiltered one is determined. The reference-corrected iodine-filtered spectrum is multiplied with this factor, resulting in the weighted iodine-filtered spectrum Fig 2D. Subtracting it Fig 2D from the unfiltered spectrum Fig 2A, the high-energy spectrum Fig 2E is obtained:

$${Image}_{high}={Image}_{unf}-a∙{Image}_{iod}$$ (2)

with *a* being the weighting factor for the iodine-filtered image.

The same procedure is performed to obtain the low-energy spectrum, but this time scaling the peak of the unfiltered spectrum to the remaining high-energy part of the matched filtered spectrum (visible in Fig 2B and Fig 2D as a little shoulder at bottom of the right slope of the peak) and calculating its ratio *b*. The weighted unfiltered spectrum Fig 2C is generated with this ratio in the same way as for the weighted iodine-filtered one. It is subtracted from the weighted iodine-filtered spectrum Fig 2D, producing a low-energy spectrum Fig 2F where the high-energy part of the spectrum is completely canceled out:

$${Image}_{low}=a∙{Image}_{iod}-b∙{Image}_{unf}$$ (3)

with *a* and *b* being the weighting factor for the iodine-filtered image and the unfiltered image, respectively. Finally, the desired difference image is obtained by subtracting the logarithmized low-energy image from the logarithmized high-energy one:

$${Image}_{KES}=\log{(Image}_{high})-\log\left({Image}_{low}\right).$$ (4)

To eliminate highly absorbing structures such as aluminum in the phantom or bones of the coastal arch in the subtraction, an empirical energy correction was applied based on the energy dependence of the attenuation coefficient. In the employed energy range, the attenuation is mainly dominated by the photoelectric effect. The resulting E^-3^ dependence away from absorption edges of aluminum and bone is exploited to highlight only regions containing contrast agent by scaling the logarithmic low-energy image with the third power of the ratio of the mean energies of low-energy and high-energy spectra obtained in the aforementioned subtraction:

$${Image}_{KES}=\log{(Image}_{high})-{\left(\frac{E_{mean,low}}{E_{mean,high}}\right)}^{3}∙\log\left({Image}_{low}\right)$$ (5)

with *E*_*mean*,*low*_ and *E*_*mean*,*high*_ being the mean energies of the low- and the high-energy images, respectively.

Due to the post-processing needed to obtain the KES image, the noise level in the resulting image will be increased in comparison to the noise level of the initial images. The variance (i.e. noise level) of the KES image can be calculated by inserting Eq (2) and (3) into Eq (5) and applying the standard propagation of uncertainty, so that it becomes:

$$\sigma_{KES}^{2}={\left(\frac{\partial {Image}_{KES}}{\partial {Image}_{unf}}\right)}_{{\bar{I}}_{unf}}^{2}∙\sigma_{unf}^{2}+{\left(\frac{\partial {Image}_{KES}}{\partial {Image}_{iod}}\right)}_{{\bar{I}}_{iod}}^{2}∙\sigma_{iod}^{2}$$ (6)

with $\sigma_{KES}^{2},\;\sigma_{unfiltered}^{2}$ and $\sigma_{iodine}^{2}$ being the variances of the KES image, unfiltered image and the iodine filtered images and the derivatives being evaluated for the mean values ${\bar{I}}_{unf}$ and ${\bar{I}}_{iod}$ of the regions of interest, that is

$${\left(\frac{\partial {Image}_{KES}}{\partial {Image}_{unf}}\right)}_{{\bar{I}}_{unf}}^{2}={\left[\frac{1}{\left({\bar{I}}_{unf}-a∙{\bar{I}}_{iod}\right)}+\frac{b∙c}{\left(a∙{\bar{I}}_{iod}-b∙{\bar{I}}_{unf}\right)}\right]}^{2},$$ (7)

$${\left(\frac{\partial {Image}_{KES}}{\partial {Image}_{iod}}\right)}_{{\bar{I}}_{iod}}^{2}={\left[\frac{a}{\left({\bar{I}}_{unf}-a∙{\bar{I}}_{iod}\right)}+\frac{a∙c}{\left(a∙{\bar{I}}_{iod}-b∙{\bar{I}}_{unf}\right)}\right]}^{2},$$ (8)

with $c={\left(\frac{E_{mean,low}}{E_{mean,high}}\right)}^{3}$. For example, the variances in regions of interest (ROIs) in the images of the heart with coastal arch are 8.8 x 10^−8^ in the unfiltered image, 4.1 x 10^−7^ in the iodine filtered image and 5.8 x 10^−3^ in the KES image. However, there is usually an increase in CNR for the structures of interest despite this increase in noise.

### Contrast to noise ratio (CNR) analysis

**Fig 3:**
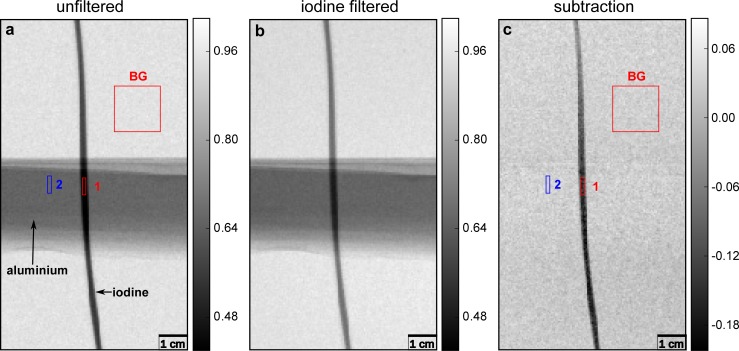
**Raw input data (a,b) and processed output image (c) associated with K-edge subtraction (KES) imaging, for a phantom containing aluminum and iodine. (a)** Unfiltered reference, **(b)** iodine- filtered image, and **(c)** KES image. Both, **(a)** and **(b)**, still exhibit aluminum overlaying the tube with iodine contrast agent. KES-imaging (for details see text) provides the aluminum-free pure iodine contrast image **(c)**. The ROIs chosen for the calculation of the CNRs of iodine (1) and aluminum (2) are shown in image **(a)** and **(c)**. The gray scales for the unfiltered and filtered images display the relative intensity / transmission of the X-ray beam, while the gray values in the KES image **c** show the negative differences in the absorption.

**Fig 4:**
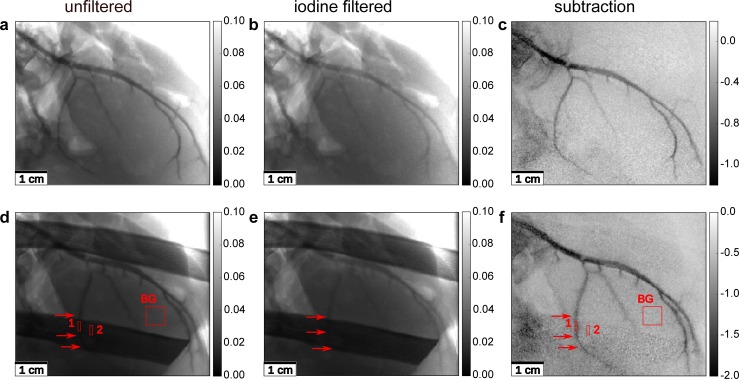
**K-edge subtraction imaging of the left anterior descending artery of a porcine heart with injected iodine contrast agent and inhomogeneous background structures**: **First row:** In both, the unfiltered **(a)** and the iodine-filtered **(b)** image, air artefacts and inhomogeneous absorption make the identification of small blood vessels difficult. In the KES image **(c)**, only the contrast agent remains visible; **Second row:** The overlying bone structures make the identification of the small blood vessel (marked by the red arrow) impossible in images **(d)** and **(e)**. Only KES imaging, combined with an energy correction, restores contrast for these blood vessels. Although in areas of lower counting statistics the image quality is compromised in the KES image **(f)** by noise, the visibility of the small vessels is increased. The ROIs for the calculation on the CNR of the blood vessel (1) and the bone (2) are shown in the unfiltered **(d)** and difference **(f)** image. The gray scales of the images were chosen such that the contrast for the coronary arteries is optimized. The gray scales for the unfiltered and filtered images show the relative intensity of the X-ray beam, while the gray values in the KES images show the negative differences in the absorption.

Two ROIs were selected for evaluating the CNR in order to investigate how the contrast to noise between the iodine contrast agent and aluminum change. The used ROIs are highlighted in Fig 3, their size was 14 x 3 for iodine and aluminum, and 33 x 33 for the background region. In Fig 4, ROIs were selected with 7 x 2 pixels for the iodine contrasted blood vessel and the rib bone and 30 x 33 for the background region.

The CNR was calculated according to the definition:

$$CNR=\frac{{|S}_{A}-S_{B|}}{\sqrt{\sigma_{back}^{2}}},$$ (9)

where *S*_*A*_ and *S*_*B*_ are the average signals in the two ROIs which should be compared and *σ*_*back*_ is the standard deviation within a larger ROI located in the background region.

### Ethics statement

The porcine heart and costal arch were obtained from a local butcher’s shop. Therefore, there was no need of an ethical approval of our experiments.

## Results

### K-edge subtraction imaging of an aluminum-iodine phantom

X-ray projection images of the aluminum-iodine phantom are displayed in Fig 3. The iodine tube and the aluminum foil are clearly visible in both the reference image and the iodine-filtered one. Only K-edge subtraction, together with the described energy correction procedure, removes the aluminum while the iodine tube remains visible. Yet, areas where aluminum was located are still visible, as the noise in these regions is increased in comparison to the empty background. This is due to the high absorption of the aluminum, which results in a lower photon statistic and thus higher photon noise. As the absorption of the aluminum depends on its thickness, a series of experiments with different aluminum thicknesses was performed to evaluate the gain in visibility dependent on the thickness of the aluminum. Table 1 shows how the CNR between the iodine contrast agent and the aluminum changes in the unfiltered and KES image with the thickness of the aluminum. It can be seen that the CNR is reduced in the KES image when the aluminum thickness is low, as the gain in contrast is not higher than the increase of the noise level.

**Table 1:** CNR values calculated between iodine and aluminum for different thicknesses of aluminum. It can be seen that starting from a certain background absorber level, the CNR in the KES image is increased. The equivalent bone thicknesses were calculated with data adapted from the XMuDat software [22].

Aluminum thickness (cm)	CNR in unfiltered image	CNR in subtraction image
0.1 (= 0.11 cm bone)	39.00 ± 1.54	24.03 ± 1.54
0.2 (= 0.23 cm bone)	27.67 ± 1.01	21.14 ± 2.94
0.5 (= 0.56 cm bone)	11.13 ± 0.58	19.02 ± 2.61

Only when the absorption in the aluminum is high, leading to a low contrast between iodine and aluminum in the unfiltered image, an increase in CNR can be reached by applying KES. This is achieved in the experiment using 0.5 cm of aluminum where the CNR increases from 11.13 ± 0.58 in the unfiltered image to 19.02 ± 2.61 in the KES image.

### K-edge subtraction imaging of a porcine heart

Fig 4A–4C shows X-ray images radiographs of the left anterior descending artery (LAD) of a porcine heart. In both the unfiltered images and the iodine-filtered ones, the coronary artery with some smaller branching vessels is visible. The iodine-filtered image was taken with a two times longer exposure time to account for the lower photon flux after filtering. The visibility of the coronary arteries is limited in both images due to overlying structures of the heart muscle and inhomogeneous absorption in the sample. In contrast, the KES image exhibits only the blood vessels, though the noise is increased. Nevertheless, even small vessels are easily recognizable, although the image quality is slightly compromised by some contrast agent leaking outside of the vessels.

Finally, a porcine costal arch was placed in front of the heart (Fig 4D–4F) to mimic a more realistic setting, where blood vessels are overlaid with bones, further reducing the vessel contrast, especially of the small vessel indicated by an arrow in Fig 4D. After KES with energy correction, only the blood vessels remain visible, reproducing the phantom results in a realistic application. This especially improves the visibility of the small blood vessels such as the small descending vessel in the lower central area of image Fig 4D (marked by red arrows), which is completely hidden behind the bone in the conventional image on the left and only visible in the KES image on the right. KES allows to level out inhomogeneous absorption and to remove uncontrasted tissue structures from the subtraction image.

As X-ray transmission through bones is low at an X-ray energy of 33.49 keV, the noise level is significantly higher in regions where the bone is located. The CNR between the blood vessel behind the rib and the rib bone was calculated to 0.44 ± 0.52, and therefore the signal lies below the noise level for the unfiltered image, and 5.16 ± 0.53 in the KES image. This supports the visual impression of better visibility of the small blood vessel hidden behind the rib bone.

## Discussion

The results of these proof-of-principle experiments demonstrate that the quasi-monochromatic spectrum of the MuCLS enables filter-based K-edge subtraction imaging.

It has previously been demonstrated by Eggl et al. [17] how image quality, contrast agent concentration and scanning time can benefit from the quasi-monochromatic high flux of a compact synchrotron. The potential improvement was first demonstrated for the actual experimental setting and the transfer of those benefits was proven via simulation with realistic clinical parameters [17]. However, in those experiments the heart was considered as an isolated object on a flat background. Here, we present a method which exploits the proven beneficial characteristics of the Munich Compact Light Source while dealing with the realistic anatomy of the heart inside the rib cage.

While the global noise level is generally compromised by the subtraction of two noise afflicted images, the local CNR of an iodine filled vessel is improved by the subtraction of strong absorber materials like the costal bones. Our experiment with a porcine heart and a costal arch demonstrates that KES strongly improves the visibility of small blood vessels, which are otherwise undetectable in the conventional image.

KES has been performed at synchrotrons in several studies. It has been shown that KES is beneficial for moving organs [5], can help with reduction of the iodine concentration in angiography and reduce the risk for the subject through transvenous injection [8,9]. KES thereby provided improved image quality for low contrast agent concentrations in comparison to clinical DSA in patient studies [8]. E. Eggl et al. have already shown in simulations that there is a significant improvement in image quality for the MuCLS in comparison to a conventional source for coronary angiography without subtraction [17]. Our method allows the separation of iodine and bone while at the same time improving the visibility of small blood vessels, while the benefits of the source compared to a conventional one remain the same. A comparison of KES and temporal subtraction (DSA) at a synchrotron by Elleaume et al. [5] showed that for small phantoms both methods show similar results, yet for larger i.e. head phantoms temporal subtraction is advantageous due to the high absorption in KES at 33 keV. The issue of the relatively low energy of the iodine edge also used in this study is further discussed below. KES can also be performed at a conventional, polychromatic X-ray source making use of a Ross filter system or a multi-energy photon-counting detectors. With a Ross filter system the spectrum can be filtered/shaped such that only the parts of the spectrum directly below and above the K-edge remain for imaging [26]. The advantage of this method is that the filter pairs provide a very well-defined and sharp energy window [27]. Yet, a large amount of the X-ray flux is absorbed in the filters reducing the statistics in the images [28]. Until now, only very simple phantoms have been measured using this method, further development is therefore needed. Multi-bin energy photon-counting detectors can also be used to obtain images above and below the K-edge of a contrast agent by discriminating X-ray photons by their energies. However, these detectors usually have an energy threshold resolution of 1–2 keV [29,30], which limits the ability to image directly around the K-edge of a material, especially when using a quasi-monochromatic X-ray source such as the MuCLS. KES with polychromatic spectra is not commonly used in clinical practice, but one approach has been made to use the technique for contrast-enhanced spectral mammography [31,32]. However, the proprietary post-processing of the images taken in this procedure is unknown so that a comparison to other imaging methods is not directly possible.

Clinical DSA uses a masking image acquired directly before the contrast agent injection to subtract the background. This procedure is prone to have artifacts arising from the movement of the patient between the masking image, the injection of contrast agent and the second X-ray image. This significant time delay between the two images required for DSA can be overcome in filter-based KES imaging as the whole data acquisition can be performed with the contrast agent already injected as demonstrated here. This makes the technique feasible for general angiography of not or only slowly moving body parts. In case of the heart, the rapid movement during a heart cycle would have to be handled. The MuCLS has a similar flux as a conventional rotating anode source as it is used in the clinical routine but provides a very narrow spectral bandwidth. Being able to tune the spectrum of the MuCLS directly to the K-edge of the contrast agent enables filter-based KES imaging with an effective switching below and above the K-edge. In this study, the time limiting factor was the manual switching of the filter. A liquid filter was used to provide an easily reproducible large-area homogeneous filter. For future studies, a solid iodine filter will be needed to enable filter changes in the order of milliseconds, which is easily possible when motorizing such a filter with standard opto-mechanical components. The idea of oscillating the beam energy of the source itself, as mentioned in [17], is also feasible but has some drawbacks: On the one hand, operating the machine in this non-standard fashion compromises its overall stability and thus the achievable image quality. On the other hand, a change of energy requires a simultaneous adjustment of about 60 machine parameters, which in some preliminary tests of this approach took in the order of 5 s for a small energy step. However, the sub-second energy changes required to cope with moving organs will not be possible this way, but can only be implemented with a rapid filter change. To cope with artifacts arising from cardiac motion and respiration, a gating of acquisition could be implemented with an electrocardiogram triggering or a ventilator machine to control breathing during image acquisition, for example.

Nevertheless, the dose applied in angiographic KES-imaging with iodine contrast agent limits its applicability in a clinical setting, due to the strong attenuation of X-rays in the human body at X-ray energies of ~33.5 keV. This drawback can be overcome by replacing the iodine-based contrast agent with a gadolinium-based one, which has already been studied for coronary angiography [33–35], especially for patients with severe iodine allergies. As the gadolinium K-edge, located at ~50.2 keV, is above the current maximum X-ray energy (35 keV) of the MuCLS, this proof-of-principle study was performed with an iodine contrast agent. However, there is no general physical limit on the achievable X-ray energy of inverse-Compton sources. Several research projects developing such sources aim at even higher X-ray energies than 50 keV, e.g. ThomX [36] and STAR [37], utilizing higher electron energies and larger storage rings. Even with the same footprint as the MuCLS, higher X-ray energies are, in principle, accessible by decreasing the laser wavelength. This ongoing evolution of compact synchrotron sources will provide the basis for dose compatible KES imaging in the future.

In conclusion, the concept of quasi-monoenergetic angiography has been extended by a K-edge subtraction method. By that, the beneficial characteristics of a compact synchrotron source are accessible in a pre-clinical setting with a non-flat background. We believe that this method can become an important tool in pre-clinical research.

## Supporting information

S1 Fig Workflow used for calculating a K-edge subtraction image at the MuCLS containing the original images. (EPS)

S2 Fig Images of aluminum phantoms with different thicknesses of aluminum. The first row shows the images taken with an aluminum strip of 0.1 cm thickness, the second row 0.2 cm and the third row The gray scales show the relative intensity/transmission of the X-ray beam. In the KES Image of the 0.5 cm aluminum strip, the strip moved between the unfiltered and filtered image so that the edge of the aluminum stays visible in the KES image.
(EPS)
